# The Economics of Filmed Entertainment in the Digital Era

**DOI:** 10.1007/s10824-021-09407-6

**Published:** 2021-04-21

**Authors:** Thorsten Hennig-Thurau, S. Abraham Ravid, Olav Sorenson

**Affiliations:** 1 University of Münster, Münster, Germany; 2 Syms School of Business, Yeshiva University, New York, USA; 3 Anderson School of Management, UCLA, Los Angeles, USA

## Digitalization and the film industry

The film industry rarely involves film anymore. The cameras and microphones use sensors. They translate the images and sounds into bits and bytes. Directors and editors manipulate the raw footage on computers, rather than with light boxes and scissors. Finished “films” get distributed as large files rather than as giant spools. Analog has given way to digital.

Although the film industry has witnessed many technological changes—the introduction of sound, of color, the invention of television—digitalization, more than any of these others, has unleashed a radical transformation of the industry. It has changed not just the nature of production, but also the businesses of distribution and of exhibition. It has challenged decades-long industry rules and routines.

COVID-19, if anything, has accelerated this transformation. When the pandemic kept people home, streaming services came to the rescue, providing audiences with filmed entertainment on their televisions, computers, tablets, and other digital devices. Since the advent of digitalization in the late 1990s, some of the more radical reconfigurations of the industry had been delayed by those with entrenched interests in the old system. But the pandemic has swept their objections aside.

Major players have repositioned themselves, re-envisioning their business models. After nearly a century of reaching audiences through middlemen, Disney became the first studio to offer their content direct to the consumer. As of December 2020, only one Hollywood studio (Sony) has yet to launch its own streaming service. Hollywood studios and their parent conglomerates have even begun to premiere their movies on streaming platforms. Universal/Comcast’s “Trolls World Tour” first played in video-on-demand. It made almost $100 million in pay-per-view, with the lion’s share going straight to the studio (Whitten 2020). Warner/AT&T announced that their entire slate of blockbuster productions in 2021 would open on the firm’s streaming service, HBO Max, on the same dates that they began to play in theaters.

Studios and theaters are not the only players affected by digitalization. The traditional model for television—often referred to as “linear” television—where a single uniform signal goes out to all viewers, mixing content with advertisements, has also been in decline. Digital distribution has made it easier and cheaper to offer the content each viewer wants, when they want it. Streaming services, such as Netflix and Amazon Prime, offer all-you-can-view buffets of video content, all with little or no advertising.

Digitalization has also been disruptive to academic research on the film industry. Decades of insight into the factors behind the success and failure of filmed entertainment had been garnered from studying the old model, one that had been dominant for more than half a century.^1^ But as that model evolves, many of the patterns that had been found no longer hold. Film has become a dichotomous business, of tentpoles and niche titles. Anything in-between hardly exists anymore. People consume more video entertainment than ever, but they do so in different ways. The strategic landscape has shifted, forcing changes across all segments of the industry’s value chain.

But exactly how all of these changes have rewritten, or will rewrite, the “recipes” for success in film has been an open question. We therefore asked a group of experts on the industry, the “Mallen Group”—economists, finance, marketing, and strategy scholars—to think deeply about how digitalization would alter the various stages of the value chain in film. Which of the past patterns can we expect to persist? Which have become obsolete? We organized the groups to cut across disciplines and to include both academics and practitioners, to ensure that each group had a broad base of expertise and grounding in the phenomenon it was studying.

This special issue of the *Journal of Cultural Economics* reports their answers. It includes five articles, each addressing the challenges that digitalization brings for filmed entertainment in a different segment of the industry: for film producers, for integrated film studios and conglomerates, for theatrical exhibitors, for television broadcasters, and for digital streaming services and platforms.

In this introduction, we begin by providing some historical background on the “Mallen Group” and its founder, Bruce Mallen. We then highlight some of the key ideas raised in these five papers. Finally, we broaden the perspective and speculate as to what the future may hold beyond what the contributing authors propose, both in terms of the continuing evolution of the industry and in terms of an emerging research agenda for this new digital era.

## A brief history of the “Mallen Group”

In the fall of 1999, Bruce Mallen, a former marketing scholar with a fascination for filmed entertainment and then Dean of the Florida Atlantic University's College of Business, convened the first of a series of conferences on the business and economics of the film industry in Boca Raton, Florida.

The original conference and its annual sequels have had many unusual features. Most noticeably, the early events brought some of the glitz of Hollywood to the academic world. Stretch limousines would transport the participants to and fro. In an homage to the Oscars, black tie ceremonies, replete with poster-size framed front pages from the articles written by the prize winners, would honor the recipients of the Carol & Bruce Mallen Award (for lifetime contributions to the study of the business and economics of the film industry). Scholars received this recognition at the awards ceremony of the Fort Lauderdale International Film Festival.

Just as streaming premieres have replaced extravaganza events in the industry, that early glamour has faded. But two more important aspects of the conference have endured over the 22 conferences that have followed. First, “Mallen Conferences” have always drawn a diverse and interdisciplinary crowd—scholars from economics, finance, history, law, management, marketing, sociology, and strategy. The common element has been the setting, the fact that all have been studying the film industry. While the cast of characters has changed over the years, the community remains united by a love of film and by an openness to diverse perspectives.

Second, the conference has been connected to practice in a way that has been unusual for academic events, even in business-related fields. Industry executives have not only attended the conference, but have also had speaking roles, as presenters, as discussants on the academic papers, and as panelists on a variety of topics. Their inclusion has helped keep the research coming out of the conference relevant to the industry and true to its institutional details.

Both unusual features of the conference reflect the unusual path that led to the first conference. Bruce Mallen began his career progressing through a series of degrees and a sequence of positions in classic academic-tale style, becoming a full professor at Sir George Williams University in Montreal (now Concordia University) before turning thirty. But then the plot twisted: Always a cinephile, Bruce left his academic appointment and Canada, moving to Southern California to make movies. He ended up producing four feature-length films. Although none of them made it to their year’s Top-10 lists, they left Bruce with deep connections to the film industry. In 1996, he returned to academia, as a Dean at Florida Atlantic University. There, he founded the DeSantis Center for Motion Picture Industry Studies, which hosted the early conferences.

Over the years, scholars from Harvard, Yale, Stanford, and dozens of other leading institutions in the USA, Europe, Australia, and Japan have participated in the conference. Papers presented over the years have been published in a diverse array of prominent journals, including *Management Science*, the *Review of Financial Studies*, the *American Sociological Review*, *Administrative Science Quarterly*, the *Journal of Marketing*, *Journal of Business* and *Organization Science*. Many “Mallen” papers have also appeared in the *Journal of Cultural Economics*, and at least three have won its prestigious Pommerehne Prize.

Bruce Mallen retired from leading the conference in 2012. Almost two decades later, after conferences at UCLA, Yale, Yeshiva University, New York, and NYU, Film University Babelsberg and Münster University co-hosted the 20th rendition of the Mallen Conference in Potsdam and Berlin in the fall of 2018.^2^ A portion of that jubilee event’s agenda organized the participants into working groups and charged them with considering the various ways in which digitalization has been transforming the industry. After two years and several rounds of revisions, five of those working groups produced papers appearing in this special issue of the *Journal of Cultural Economics*. These papers bring a scholar’s lens to the multifaceted and radical changes that digitalization has brought to the film industry, assessing the factors that separate our digital era from its analog predecessor.

## The contributions

The five papers featured in this special issue address the same phenomenon, but study its impact on different segments of the industry. Historically, companies in the film industry in the USA have operated in only one of these segments. In part, that reflects the long-lasting implications of the Paramount decision, an antitrust settlement that required film studios to divest from theaters (Conant 1960). But in part, it also reflects some of the underlying economics of these segments (Caves 2000).

But the relatively clear separation of activities has been under threat from digitalization. In some cases, distributors have moved into the production of content. Witness Netflix, Amazon, and now also Apple developing their own “originals”—series, movies, and shows distributed, often exclusively, over their own streaming services. Content producers, meanwhile, have been diversifying downstream. These efforts began as collaborative efforts. Movielink, for example, offered video-on-demand from the libraries of all of the studies (Hennig-Thurau & Houston 2019). But increasingly the studios and their parents have chosen to go it alone. Disney actually cut its lucrative ties with streaming services to offer its content exclusively via its own streaming service.

**Fig. 1:**
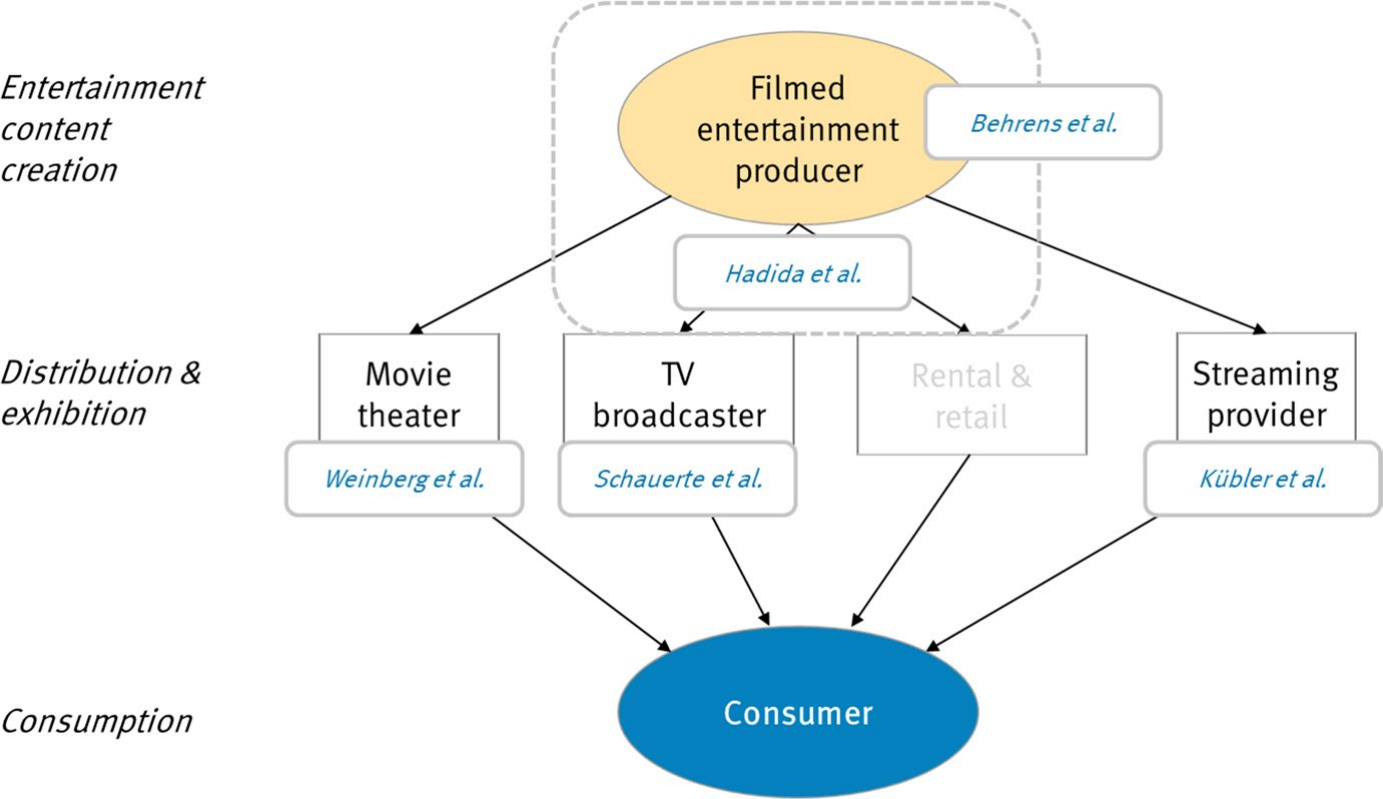
Value creation in filmed entertainment and structure of the special issue

Figure 1 illustrates the traditional roles that firms have played in creating and distributing filmed entertainment and the relationships of the articles in this special issue to these roles. Behrens et al. (2021) take the perspective of an individual film producer, Hadida et al. (2021) of an integrated film studio that produces not just a single film but an annual slate of them. Weinberg et al. (2021) study theater owners, and Schauerte et al. (2021) analyze television broadcasters. Kübler et al. (2021) complement these perspectives by studying one of the challengers of the digital age, subscription platforms, putting a focus on their content valuation strategies.

Let us being with production. In the film business, putting together a hit movie has always been considered more art than science. The story, the director’s vision, the performances of and chemistry between many actors and actresses, the cinematography, and the music all matter. Having a hit, however, depends not just on these individual elements but also on whether they fit together (De Vany & Walls 1996).

Behrens et al. (2021), however, describe the many ways in which the digital revolution, particularly in terms of the availability of granular viewing data, has begun to interject more “science” into this production process. Information on what people watch, on where in the movie or serial they stop watching it, and on what people watch next provides producers with a far better picture of the demand side. Combined with a variety of analytic techniques, consultants have been developing better ways of analyzing plot concepts and scripts before they get selected and finalized, talent before actors and actresses get cast, audience fit before engaging in marketing campaigns, and expected sales before exhibition. Interestingly, platforms, such as Amazon Prime and Netflix, potentially have an advantage in developing and using these tools as they collect and control access to much of the relevant data.

Hollywood “studios” also produce films, but their perspective differs from that of individual producers in terms of scale and scope. On the scale side, studios produce portfolios (or slates) of films. They often allocate billions of dollars to “franchise” films (e.g., Spider-Man or Harry Potter), expensive productions beyond the reach of most individual producers. Those “tentpole” productions have been found to have higher odds of success. But they are not fail-proof. On the scope side, in addition to producing content, studios also promote films and contract with theaters, television stations, and streaming services for the delivery of content to the consumer (Vogel 2020).

Hadida et al. (2021) argue that the digital age has challenged the core institutional logic for these studios. Institutional logic refers to a way of thinking about the problem. One firm, for example, might focus on minimizing production costs, whereas another might try to maximize customer satisfaction. For decades, the Hollywood studios have operated with an eye to maximizing box office performance, in other words ticket sales in theaters. That has governed their choice of release dates, the way in which they advertise and promote films, and even the terms in the contracts they sign with producers, television stations, and streaming services. Hadida et al. label this approach a “commitment” logic.

By contrast, streaming services have adopted a “convenience” logic. They have almost all converged on a subscription model. Most of what they do, therefore, has been in the name of increasing subscriptions. This logic has led these services to accumulate large libraries of films and television shows, even though many of these offerings have limited appeal. It has led them to adopt evaluation and recommendation systems to help subscribers find additional content. And most recently, it has led them to develop their own proprietary content to attract more users to their platforms.

Which “logic” will win? Hadida et al. (2021) discuss a number of potential scenarios. Some sort of blending seems highly likely. Studios, for example, may embrace the analytics described by Behrens et al. (2021) but continue to focus on big-budget films designed for the big screen. Streaming services, meanwhile, will probably continue their forays into the production of content and begin to release some of these films in theaters, perhaps even contracting with the studios to handle the distribution.

Theaters, as exhibitors of movies, have traditionally been the main setting in which people have consumed films (Vogel 2020). But that has been changing. People increasingly watch filmed entertainment not just on their televisions but on a variety of mobile digital devices. COVID-19 has, at least temporarily, accelerated this trend. Movie theaters, in response, have been adapting, adopting their operations to differentiate further the experience of the big screen from that offered by streaming to digital devices.

Weinberg et al. (2021) discuss opportunities that digitalization offers to theater owners. Online sales can smooth the purchase process. Loyalty programs can help them to provide more targeted pricing and promotion, again revealing the importance of analytics (cf. Behrens et al. 2021). But digitalization also enables more subtle adaptations. Digital projectors, for example, allow multiplexes to allocate firms to individual theaters at a moment’s notice. When they have multiple theater sizes available, multiplexes can better match supply to demand, improving their capacity utilization.

As another major distribution channel of the analog age of film, “linear” television, initially broadcast over the air, later piped through cables, has enjoyed a long run of success. In the traditional model, this success has relied on selling the attention of viewers to advertisers. Consumers pay for programming with their time. But digitalization increasingly allows viewers to watch what they want, when they want, fast-forwarding through the commercials. Advertisers consequently have been allocating less and less of their budgets to the medium. Although linear television has not disappeared, it represents a shrinking share of filmed entertainment (Vogel, 2020).

Schauerte et al. (2021) consider the options linear television firms have to adapt to the digital age. Should they (1) remain linear-only television companies; (2) combine the linear offering with advertising-based video-on-demand services; (3) combine the linear offering with subscription-based video-on-demand services, or (4) combine the linear offering with both kinds of video-on-demand services. Building on the resource-based theory of the firm, the authors examine which sets of market-oriented and internal strategic resources each strategic option requires. Interestingly, as much as Hadida et al. (2021) argue that we should expect a blending, Schauerte et al. (2021) argue that linear television can remain relevant in the digital age, by leveraging synergies between linear and video-on-demand services.

In the final article of the special issue, Kübler et al. (2021) evaluate the strategies of digital subscription platforms. Hadida et al. (2021) called attention to the differing logics of these platforms, oriented toward subscriber growth and retention. This article explores the strategic implications of that logic. Treating the platforms as a form of product bundling, they provide a framework for assessing the value of each piece of content. Content can create value in a variety of ways, by bringing consumers to the platform, by retaining them, or by providing opportunities for advertising or cross-selling. Understanding that perspective can help traditional firms understand the streamers’ logic, perhaps smoothing future cooperation, or allowing them to manage better their own streaming services.

## Some disciplinary perspectives

This final section of our editorial considers what questions we expect to motivate future research, given this digital transformation. It provides a bit of a research agenda.

We intentionally organized the working groups around industry segments and business models, and the chapters follow that focus. But most “Mallen Group” members identify also with a discipline, and a large share of past research on the film industry has been published in disciplinary journals. We expect that these disciplinary lenses will continue to direct research attention, so we have organized our outlook in terms of three broad groups of scholars: economics and finance, marketing, and organization theory and strategy.

But before we put on the disciplinary lenses, let us highlight some fundamental changes, and challenges, that digitalization imposes on *all* scholars of filmed entertainment. The attractiveness of film as a research setting stemmed in part from being able to treat each film as an independent project and product with a relatively clear measure of performance—most of the revenues either came from or have been highly correlated with box office sales.

The rise of streaming raises multiple issues. More and more of the content being produced appears in serial form, meaning that researchers cannot treat the projects as independent or easily isolate the performance of an individual project. Also, streamers control access to viewing data and have so far displayed limited interest in opening it to partners, including researchers. Success, itself, has also become a fuzzier concept, as individual productions can create value for a subscription-based platform in so many ways (e.g., Kübler et al. 2021).

Lest scholarly readers’ despair, digitalization also offers many research opportunities. Streaming services amass an unprecedented amount of information on the consumption of filmed entertainment, and companies such as Filmchain—a budget and revenue management service based on blockchain technology—allow fine-grained monitoring of cash flows from exhibitors to rights holders. Should these companies cooperate with researchers, we could greatly improve our understanding of what, why, and how people consume filmed entertainment, to the benefit of both science and the platforms. To avoid becoming irrelevant as film scholars, we must accept that digitalization also changes *our* world and adapt our data, methods, and topics to the new realities of the industry.

### Finance and economics

One of the fundamental questions from a finance and economics perspective has been the risk and return characteristics of the film industry. Films have historically been a business of hits and misses, with far more misses than hits. Because of the highly skewed nature of the returns, these hits account for the vast majority of the profits in the industry (De Vany & Walls 1996).

Much research has therefore focused on the nature of the risk-return trade-off in the industry (e.g., De Vany & Walls 1999, 2002; Palia et al. 2008; Ravid & Basuory 2004). One of the most consistent findings, dating back to Ravid (1999), has been that sequels and franchises (but not remakes) may be the holy grail of the industry, with projects featuring lower risk *and* higher return (see also Bohnenkamp et al 2015; Filson & Havlicek 2018; Palia et al. 2008). Filmmakers can use information from the sales of a successful early film to decide whether or not to produce the next one, thereby exercising a real option.

Streaming platforms appear to have learned this lesson. As they moved into production, they have focused more on creating serialized content than on stand-alone movies. But streaming platforms often commit to producing an entire series upfront. That has advantages in terms of reducing the costs of production. But it also carries a price-tag: producers cannot capitalize on the information gained from an earlier installment to determine whether or not to produce the next one, losing the real option value of serial content in the process.

Production companies and studios that sell movies outright to streaming platforms also transfer both the risk of failure and the benefit of success to a platform. This arrangement will change the risk and return from their point of view. It will probably affect the mix of movies studios would like to produce. High-risk blockbusters, for example, may seem less attractive. Platforms may dictate the types of movies that they will buy (this already happens to some extent), but platforms have a very different risk profile. They may opt for sure-bet crowd pleasers, but, as discussed in Kübler et al. (2021), platforms can potentially profit also from films even if they appeal strongly only to a niche audience.

One effect of these decisions has been a blurring of the boundaries between made-for-television movies, television series, and theatrical movies. Theatrical movies have been differentiated from other forms of filmed entertainment. Theatrical movies have had larger budgets, and higher production values, than TV productions and were intended for the big screen. However, current serialized content often has the same production budgets and values once reserved for the theater.

These changes also have implications for career paths. Research on the careers of actors, directors, and producers has usually focused on one mode of production, since different types of projects had different and distinct characteristics (e.g., Baker & Faulkner 1991; Faulkner & Anderson 1987; Han & Ravid 2020; John et al. 2017; Zuckerman et al. 2003). However, increasingly, talent has been moving across the various types of productions. Although these eroding boundaries will make careers more difficult to study, they may also raise a novel set of career-related questions.

Digitalization also promises to raise interesting questions about contract design and industry structure (Chisholm 1997; Filson et al. 2005; Palia et al. 2008). For instance, contracts with exhibitors will have to adjust as studios no longer have an interest in guaranteeing exhibition windows. These changes will affect the incentives and the entire business model of movie exhibition.

Another interesting aspect of contract design relates to pricing. Box office sales have traditionally been an important leading indicator of value. These sales have therefore helped set prices for subsequent channels, such as international markets and home video. Everyone had access to the same information; they agreed on its validity. But in streaming, performance data remain proprietary, introducing information asymmetry into any negotiations with other parties.

Finally, the largest question concerns the structure of the industry as a whole. The new streaming services have begun to compete on exclusive content. Will this trend continue, or will contracts allow multiple aggregators to compete over offering the same films to consumers, either on a pay-per-view basis or as part of a subscription service. The “new” film industry may therefore provide a rich environment for future research in industrial organization economics and finance.

### Marketing

Marketing scholars study the creation of value through transactions by and relationships with market partners. Consumer preferences play a central role. Those companies that can best address consumers’ preferences create the most value.

Digitalization, however, has been fundamentally changing these preferences. It has influenced the types of filmed entertainment that consumers want. It has changed the channels they favor for consuming these offerings. It has affected the prices they consider appropriate. And it has altered the ways in which and extent to which they attend to messages from marketers and from their fellow consumers. Here are some of the most pressing, yet underesearched, marketing issues that this transformation has raised.

*Creation.* Digitalization provides those who tell stories via film and those who produce them with powerful tools not only to analyze what kinds of narratives work (see Behrens et al. 2021), but also to visualize these stories. Digital technologies can bring beloved characters, heroes, and villains to the screen in unprecedented ways. Consider the creation of novel footage of the deceased Carrie Fisher’s Princess Leia from bits and bytes stirred with some machine learning. The technical possibilities seem limitless. But who owns the rights? And how will consumers’ react? So far, audiences appear torn between adoration and anger. Mori’s (2012) “uncanny valley” theory might serve as a powerful starting point for a better understanding of their reception.

Digitalization also offers other technological opportunities to filmmakers, such as virtual reality (VR) and interactive storytelling. VR has been a disappointment so far. But its immersive powers appear so enormous that the entertainment industry, and its scholars, should find new ways to create value with the technology. Behrens et al. (2021) suggest that the “killer app” for VR might come from combining the technology with video-game-like interactive storytelling. What business models might best fit such an application?

*Distribution.* Or will VR become a distribution channel? One could imagine VR being used to create digital venues in which we (or our avatars) meet and watch movies together—a high-tech variant of Amazon’s “Watch Party” feature. Would such a service have mass appeal that might substitute for going to the theater? More generally, how will VR-based products and services interact with the existing business models in the industry? Should industry incumbents invest in their development, despite their potentially cannibalizing effects? Or should they leave them for outsiders, such as Facebook (who owns the current VR device leader, Oculus)?

Even leaving aside the possibility of VR venues, digitalization raises other distribution-related questions. How can studios optimize their release strategies given the new plethora-of-channels environment? With the implosion of the rigid windowing model—with a clear sequencing of releases in various channels—scholars might want to direct their attention toward a contingency approach for film distribution.

*Pricing.* Discredited by the “nobody knows anything” mantra of the analog era, pricing has played only a supporting role in film research. But the rise of subscription models, with bundling becoming dominant, may allow pricing to headline. Will single purchases persist in a world dominated by subscriptions? How should studios price “premium” video-on-demand offerings, which can generate incremental revenue but potentially at the expense of the value of the subscription bundle? After charging extra for Mulan, Disney released Soul as part of their basic fee. Under what conditions can video-on-demand still add value to consumers—and firms?

*Communication.* Scholars have argued that the digital environment for marketing communication resembles a pinball machine, with chaotic elements and limited firm control (Hennig-Thurau et al. 2013). How can studios reach large audiences with meaningful messages in such an environment? Research on social media sentiment and topics in the context of films has shed initial light on this issue (Kupfer 2018; Liu et al. 2018), but the ever-proliferating variety of channels (TikTok!) points to a need for more research. The difficulty of reaching audiences in this pinball environment on a limited budget has also contributed to the demise of smaller productions. Can marketing scholars find ways to use the environment that require less ad spend, resurrecting the financial viability of mid-sized theatrical releases?

### Organization theory and strategy

As in the other streams, many of the questions examined in the context of film by organization theory and strategy scholars have been ones of broad interest to that group, even beyond film.

Categories—how consumers sort products, services, organizations, and people into groups—have been a vibrant subject of study over the last two decades. One of the important ideas within that research emerged in the context of film. Hsu (2006) found that films that straddled categories (genres), such as a horror-comedy, did better at the box office but received lower average ratings from audiences. These films had broad-but-shallow appeal. Similar results have since been found in a wide variety of settings, from software to careers (Hannan et al. 2019; Zuckerman et al. 2003).

Categories and categorization will undoubtedly remain important. They may even become more so. Streaming services typically organize their offerings according to genre, so users frequently choose a category and select a film. Recommendation systems, moreover, often reinforce these classifications. But streaming also changes the incentives for producers. Offerings with an intense appeal to a niche audience become more valuable relative to those with broad-but-shallow appeal if these offerings attract and retain subscribers. Genres of films may therefore become more distinct, have “sharper contrast” in the language of the literature on categories.

Social relationships have been another active area of study. Connections have been found to influence a wide variety of outcomes in the film industry: who gets cast (Faulkner & Anderson 1987; Grugulis & Stoyanova 2012), the matching of films to distributors (Cattani et al. 2008; Sorenson & Waguespack 2006), the degree to which films get promoted (Sorenson & Waguespack, 2006), and who wins awards (Rossman et al. 2010). At first blush, the transition to digital might not seem to matter here: friends are friends, or at least the devil that you know seems better than the one that you don’t. But the importance of these connections often stems from uncertainty, particularly about quality (Sorenson & Waguespack 2006). As data availability and analytics reduce this uncertainty, these connections may matter less.

But these analytics may introduce other problems. Algorithmic bias—the idea that machine learning and other forms of data analysis may reify existing forms of discrimination—has been a hot emerging topic (Cowgill & Tucker, 2020). As these algorithms become more influential to production and distribution decisions, they could easily create new forms of self-confirming stratification in the industry (cf. Sorenson & Waguespack 2006).

In the strategy literature, the film industry has been an unusually interesting setting for studying corporate strategy, the scope and organization of the firm and its effects on behavior and performance. Vertical integration, for example, appears to buffer producers from uncertainty in distribution and exhibition and to allow exhibitors to respond more effectively to information on film popularity (Gil 2009; Hanssen 2010; Negro & Sorenson 2006). But engaging in multiple markets also strains managerial attention and organizational resources, when unexpected events affect one of these markets (Natividad & Sorenson 2015). With digitalization, this segment of the strategy literature will probably expand. As filmed entertainment firms become more varied in their scope and in the strategies that they pursue, the opportunities for exploring the performance implications of these decisions will expand, as does the need for understanding what works and what does not.

## A Happy(?) Ending

The film industry has weathered the introduction of many innovations, from feature-length films and sound, to stars and color, to competition from television and wide-release strategies. But the shift from analog to digital has had more profound and wider-ranging implications for the industry than any of these earlier innovations. Despite the fact that this digital revolution began more than 20 years ago, producers, distributors, exhibitors, and others continue to adapt to it. They have discovered and experimented with a range of business models and practices enabled by digitalization. It remains far from certain what will prevail and what will disappear.

As much as digitalization challenges the accumulated wisdom of the film business, these are exciting times for scholars of the industry. Let us embrace these opportunities, find ways to overcome the research challenges of the “new” digitalized industry, and continue our quest to shed light on, and guide, the industry’s future paths. Because one thing can be taken for granted: Humans’ hunger for filmed entertainment will persist, digital times or not!

## Footnotes

1 For reviews of this large literature, see Hadida (2009) and Hennig-Thurau & Houston (2019). For an excellent description of the industry and many of its contractual features, see Vogel (2020).

2 More about the “Mallen Group” and its conferences can be found at http://themallenconference.org.
